# The Evaluation of Postoperative Complications and Oral Health-Related Quality of Life Following Dental General Anesthesia for Early Childhood Caries

**DOI:** 10.7759/cureus.47325

**Published:** 2023-10-19

**Authors:** Mebin George Mathew, Ganesh Jeevanandan

**Affiliations:** 1 Pediatric and Preventive Dentistry, Saveetha Dental College and Hospitals, Saveetha Institute of Medical and Technical Sciences, Chennai, IND

**Keywords:** full-mouth rehabilitation, postoperative pain, postoperative complication, pediatric dentistry, dental general anesthesia, adverse life events, early childhood caries, general anesthesia, dental care for children, morbidities

## Abstract

Aim

The study aims to evaluate the postoperative complications and oral health-related quality of life (OHRQoL) following dental general anesthesia for early childhood caries (ECC).

Materials and methods

Two hundred children aged between three and six requiring full-mouth rehabilitation for general anesthesia were recruited for the study. Demographic data and oral health-related quality of life using the Early Childhood Oral Health Impact Scale (ECOHIS) were collected before the surgery. Postoperative complications and oral health-related quality of life were evaluated after 24 hours and at a one-week follow-up appointment.

Results

All 200 children returned for the follow-up appointment after one week. Pain was found to be the most common postoperative complication after 24 hours (52%) and one week (6%). The oral health-related quality of life showed a significant improvement after one week (P < 0.001)

Conclusion

Children may experience a postoperative complication after full-mouth rehabilitation, which resolves within a week. Dental pain was the most common operative complication in the present study. Oral health-related quality of life showed significant improvement after full-mouth rehabilitation.

## Introduction

Early childhood caries (ECC) refers to the occurrence of cavitated or non-cavitated lesions in children under 71 months of age [1]. A highly prevalent disease, ECC has affected more than half a billion children and profoundly impacts them, influencing the overall health of both children and their caregivers [2].

ECC starts as a white spot lesion, which is a non-cavitated carious lesion. At this stage, ECC is reversible if oral hygiene improves or if preventive dental treatment begins. These white spot lesions can cavitate quickly since the primary enamel is thin, leading to a swift progression of ECC. Children with ECC often present to dental offices with pain or abscesses and widespread dental lesions that require aggressive dental treatment [3,4]. Given that children with ECC are typically very young, they can be uncooperative and highly anxious. This can make managing their dental treatment in the office challenging. Chairside management might require multiple appointments, depending on the severity of ECC, the dental treatment needs, and the child’s ability to cooperate. To offer optimal treatment, full-mouth rehabilitation under general anesthesia is employed for children with ECC [5,6]. Today, full-mouth rehabilitation under general anesthesia is routinely used as a day-care procedure for managing ECC because it offers benefits such as high efficiency, safety, and the ability to provide high-quality treatment [4-6].

Despite high success rates with treatment under general anesthesia for ECC, only a few studies have explored postoperative morbidity. These studies have found that children experience postoperative complications, such as pain and bleeding [7-9]. Various published papers have reported postoperative complications ranging from minimal to significant. However, comparing these results can be challenging, especially considering variations in socioeconomic status, standard operating procedures for full-mouth rehabilitation, and postoperative analgesic usage from one team to another [9-11]. While oral health-related quality of life (OHRQoL) has been observed to improve after treatment, the effects of postoperative complications on OHRQoL have not yet been explored [4-6].

Several factors can contribute to the development of postoperative complications after treatment under general anesthesia. These include age, intubation difficulty, type of dental treatment, local anesthetic use, intraoperative use of analgesics, and patient and parental compliance with postoperative instructions [7,11]. Given the paucity of research on postoperative complications, this study aimed to investigate these complications and the changes in OHRQoL in a prospective cohort of children requiring full-mouth rehabilitation under general anesthesia and the factors influencing them.

## Materials and methods

This cohort study began after receiving permission from the Institutional Human Ethical Committee of Saveetha Dental College and Hospitals in Chennai, India (IHEC/SDC/FACULTY/21/PEDO/2820).

Children aged between three and six years, diagnosed with ECC and requiring comprehensive dental treatment under general anesthesia, were included. Those with special healthcare needs or those who did not have general anesthesia were excluded. Of the 621 patients diagnosed with ECC seen in the Pediatric Dentistry Division, 200 who met the selection criteria and consented to participate were recruited for the study. The parents were thoroughly informed about the project in a language they could understand and converse in. All parents had the opportunity to discuss their concerns with the pediatric dentist and anesthesiologist about the procedure and postoperative care.

A standardized treatment protocol was adopted by the operating team for all patients. Carious lesions on the occlusal surface were restored with either glass ionomer cement or a composite. Stainless steel crowns rehabilitated the primary molar teeth with carious lesions affecting multiple surfaces, while strip crowns were used for the anterior teeth with multi-surface lesions. The teeth with pulpal involvement were treated according to the guidelines of the American Academy of Pediatric Dentistry, followed by the placement of the final restoration: strip crowns for the anterior teeth and stainless steel crowns for the posterior teeth. The teeth with necrotic pulps and those deemed non-restorable were extracted under local anesthesia. Space maintainers were installed post extraction, depending on the clinical scenario. The Early Childhood Oral Health Impact Scale (ECOHIS) assessed OHRQoL on the day of the surgery, 24 hours post surgery, and during a follow-up appointment seven days post surgery. Postoperative complications were evaluated 24 hours and seven days after the procedure.

Oral health-related quality of life assessment

The ECOHIS was utilized to assess the OHRQoL. The ECOHIS is a validated and reliable tool for measuring the OHRQoL of preschool children [12]. It comprises two sections: the child impact section (CIS) and the family impact section (FIS). It has 13 questions that parents answer.

Preoperative and postoperative survey

**Figure 1:**
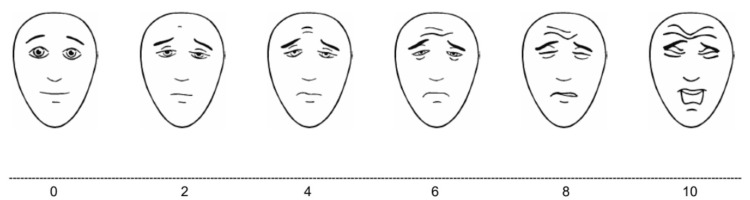
Face Pain Scale-Revised

Before initiating treatment, the Face Pain Scale-Revised (Figure 1) and a postoperative complication questionnaire were administered to the guardians, who were given opportunities to ask questions [13]. The questionnaire was completed by the parents upon discharge after 24 hours and again at the follow-up appointment one week later. It focused on postoperative complications. Children who scored greater than 4 on the Face Pain Scale-Revised were advised to take analgesics prescribed by the pediatric dentist.

Data from clinical observations and the questionnaire were input into Excel version 2019 (Microsoft® Corp., Redmond, WA) and analyzed using the Statistical Package for Social Sciences (SPSS) 23.0 (IBM SPSS Statistics, Armonk, NY). The data analysis incorporated descriptive statistics and bivariate tests. P ≤ 0.05 was deemed significant.

## Results

All participants, accompanied by their parents, presented to the department for their follow-up review appointment one week after the initial visit, ensuring a 100% retention rate at the end of the study.

Of the participants, 53% were males, and 47% were females. Mothers accompanied 70.5% of the participants. The mean age of the patients was 4.11 ± 1.47 years. Caries experience, measured by the decayed, missing, and filled primary teeth (DMFT) score, was 12.48 ± 5.61. The demographic data for this study is summarized in Table 1.

**Table 1:** Demographic details SD, standard deviation; DMFT, decayed, missing, and filled primary teeth

Parent demographics	N (%)
Relationship to the child	
Mother	141 (70.5)
Father	59 (29.5)
Education level	
Secondary or below	162 (81)
Post-secondary or above	38 (19)
Child demographics	N (%)
Gender	
Male	106 (53)
Female	94 (47)
Age (years), mean (±SD)	4.11 (1.47)
Child’s caries experience	
DMFT score, mean (±SD)	12.48 (5.61)

Treatments administered under general anesthesia are detailed in Table 2. The mean duration for full-mouth rehabilitation under general anesthesia was 118.39 ± 31.56 minutes. Stainless steel crowns and pulpectomy were the most common treatments for the participants.

**Table 2:** Summary of dental procedures done for the participants during full-mouth rehabilitation under general anesthesia

Serial number	Procedure	Children who received the treatment, n (%)	Average number of treated teeth
1	Pulpectomy	198 (99)	8.02 ± 1.79
2	Stainless steel crowns	198 (99)	8.93 ± 1.64
3	Zirconia crowns	28 (14)	2.13 ± 0.71
4	Strip crowns	26 (13)	3.61 ± 1.12
5	Restorations	38 (19)	3.04 ± 1.26
6	Extraction	46 (23)	1.33 ± 1.07

The most common postoperative complication within 24 hours was pain, experienced by 52% of patients, followed by fatigue (39%) and mastication problems (37%). The least common complication was a sore throat, reported by 0.5% of the participants. At the one-week follow-up, mastication problems (7%) remained the most common issue, with postoperative pain following at 6%. Of the 15 complications evaluated, 11 were resolved by the one-week follow-up (Table 3).

**Table 3:** Postoperative complications among the participants after full-mouth rehabilitation under general anesthesia A P-value of ≤0.05 was considered significant

Serial number	Postoperative complications	Incidence after 24 hours	Incidence after seven days	P-value
1	Postoperative pain	52%	6%	<0.001
2	Weariness	39%	2%	
3	Mastication problem	37%	7%	
4	Drowsiness	31%	0%	
5	Agitation	14%	0%	
6	Bleeding	12%	0%	
7	Fever	9%	0%	
8	Vomiting	5%	0%	
9	Nausea	5%	0%	
10	Cough	4%	0%	
11	Epistaxis	3%	0%	
12	Excitement	2%	0%	
13	Constipation	2%	0%	
14	Diarrhea	2%	0%	
15	Sore throat	1%	0.5%	

A statistically significant improvement in OHRQoL was observed both within 24 hours and after seven days (P < 0.001). The CIS scores decreased from 15.7 ± 4.1 at baseline to 12.3 ± 3.9 after 24 hours and further to 7.7 ± 1.9 at the one-week recall. All domains of CIS showed a statistically significant improvement (P < 0.001). FIS scores also significantly dropped from 9.6 ± 2.7 at baseline to 6.1 ± 2.8 after 24 hours and then to 3.5 ± 2.6 at the one-week follow-up (Table 4).

**Table 4:** Mean scores and mean differences in Early Childhood Oral Health Impact Scale (ECOHIS) scores before and after dental rehabilitation under general anesthesia A P-value of ≤0.05 was considered significant SD: standard deviation

ECOHIS domains	Number of items	Baseline (±SD)	After 24 hours (±SD)	After seven days (±SD)	P-value
Child impact section	9	15.7 ± 4.1	12.3 ± 3.9	7.7 ± 1.9	<0.001
Child symptoms	1	3.2 ± 0.9	2.5 ± 0.7	1.1 ± 0.7	<0.001
Child function	4	6.7 ± 3.2	5.3 ± 1.2	3.1 ± 1.2	<0.001
Child psychology	2	2.9 ± 1.7	2.3 ± 1.2	1.6 ± 1.4	<0.001
Child self-image and social interaction	2	2.9 ± 1.8	2.2 ± 1.5	1.5 ± 1.2	<0.001
Family impact section	4	9.6 ± 2.7	6.1 ± 2.8	3.5 ± 2.6	<0.001
Parental distress	2	4.9 ± 2.3	3.2 ± 1.5	2.1 ± 1.2	<0.001
Family function	2	4.7 ± 1.1	2.9 ± 1.3	1.4 ± 0.8	<0.001
Total ECOHIS score	13	21.6 ± 9.5	18.4 ± 5.7	11.2 ± 4.2	<0.001

## Discussion

Most children visit the dental office when their ECC has progressed to a severe stage. At this point, an aggressive form of treatment may be necessary to rehabilitate the oral cavity and restore its form and function [14]. Given that these children are young and may not cooperate for multiple treatments in a single appointment, several visits are often necessary for comprehensive treatment. This can mean prolonged hours at the dental office, multiple days off work for parents, lengthy travel times to the dental office, and potentially compromised treatment quality due to the child’s level of cooperation [2,3,7]. As a result, children with ECC are often treated under general anesthesia for full-mouth rehabilitation [5,7].

Postoperative morbidities, such as pain and bleeding, can occur with general anesthesia, depending on the treatment plan [10,11]. Dentists often have minimal contact with patients and their parents post procedure and might be unaware of various complications that can arise after surgery [7,9]. Although most postoperative complications after full-mouth rehabilitation are short-lived and minimal, they can still cause anxiety for both parents and children. This anxiety can be alleviated if the operating pediatric dentist briefs parents or caregivers about potential postoperative outcomes during the initial appointment [8-11].

The most prevalent postoperative complication in the present study was postoperative pain, seen in 52% of the patients after 24 hours. This eventually reduced to 6% at the end of one week. Pain has always been a subjective phenomenon, with perceptions varying between individuals. The best method for evaluating pain is self-reporting [15,16]. Postoperative pain in children after dental day-care surgery has been found to range from 27% [11] to 95% [16]. Ninety-nine percent of the participants required pulpectomy and stainless steel crowns. Pulpectomies have been associated with postoperative pain. Reporting post-endodontic pain has been found to be subjective, usually due to the extrusion of debris [17]. The placement of multiple stainless crowns in a single appointment can result in pain due to changes in occlusion or an increase in vertical dimension. In children, this is compensated by jaw growth and the nature of the bone, leading to the occlusion returning to normal in around seven to 15 days [18]. The number of extractions has also been identified as an etiological factor for postoperative pain [7,19]. Variations in reported rates for postoperative pain can be attributed to factors such as the age of the participants, the use or nonuse of local anesthesia, the type of dental procedures performed, and the pain assessment tools [7-11,16,20]. The reported rate in our study can be attributed to all participants being below six years of age. Their level of understanding might not be as refined as that of adults, and they may struggle to respond accurately to questions [11,20]. Another contributing factor could be the duration of the procedure. Longer surgeries are often considered more complex and may also contribute to postoperative pain [7-9,20].

Weariness was the second most commonly observed postoperative complication, reported by 39% of the participants, while 31% reported drowsiness. Weariness has been attributed to the length of the anesthetic procedure. According to Atan et al. [21], a 10-minute increase in the anesthetic procedure raises the odds of experiencing weariness by 15%. The weariness reported in the present study is less than that reported in previous studies [8,21,22]. Weariness decreased to 2% among the participants, but no instances of drowsiness were observed. A well-planned procedure, characterized by cooperation and a conducive working environment among anesthetists, pediatric dentists, and nurses, can optimize operating time, thereby minimizing the duration of surgery and the incidence of both weariness and drowsiness among participants [7,8,20,21].

Nearly 37% of participants experienced mastication issues 24 hours after the procedure. This might be due to a new occlusal relationship resulting from the restoration of multiple teeth, which rehabilitated tooth surfaces destroyed by ECC [23]. Stainless steel crowns facilitate the complete rehabilitation of occlusal surfaces, which in turn aids in improved mastication and, thus, better growth and development [18,22]. Normal occlusion readjustment typically occurs within two weeks, allowing children to eat without postoperative complications. van der Zee et al. attributed this occlusal readjustment to the nature of alveolar bone and jaw growth [18]. This may also explain the reduction in masticatory problems to 7% at the end of one week.

Agitation was observed in 14% of the children 24 hours post procedure but was absent in all children after one week. This rate is considerably lower than previously reported rates [8,24]. Factors such as separation from parents for surgery, an extended fasting period leading to hunger and thirst, and the unsettling experience of waking up surrounded by unfamiliar faces can contribute to agitation and irritability. Studies suggest that the use of sevoflurane might result in postoperative agitation and irritability [7,25].

Bleeding was observed in 12% of the patients at 24 hours. This can be attributed to the number of extracted teeth, the presence or absence of a vasoconstrictor in local anesthesia, and injury to the gingiva during crown preparation. The prevalence of bleeding in the current study was much lower than previously reported rates [9,11,20]. Although bleeding has been mentioned in multiple studies, there is a high possibility that what was observed was postoperative ooze, which might have been mistaken for bleeding by children, parents, or caregivers [8]. No bleeding was reported in any of the participants at their one-week follow-up. The use of gauze packs, absorbable gelatin sponge packs, and sutures can assist in preventing postoperative bleeding, which in turn helps in reducing anxiety for both patients and parents or caregivers [8,11].

Fever was reported in 9% of the children after 24 hours but in none after one week. The prevalence of fever in this study is similar to that reported by Needleman et al. (10%) [8] but less than that of Ghafournia et al. (45.8%) [26]. The onset of postoperative fever has been attributed to the fasting period before surgery, which can lead to dehydration and, consequently, fever [26]. The type of intubation, trauma to adjacent tissues, the use of drugs, and the presence of infection have also been considered factors in postoperative fever onset [8,20,26].

Vomiting was observed in 5% of the children 24 hours post surgery but was absent at the one-week review appointment. Nausea was reported in 5% of the patients after 24 hours and was not present during the one-week follow-up. The rates for both nausea and vomiting were much lower than those reported by Farsi et al. (26%) [22] and Ghafournia et al. (40.3%) [26]. The discrepancy in reported rates might stem from the choice of pre- and postoperative drugs used, the nature of the surgery, and dietary habits before and after surgery [26]. Opioids, such as fentanyl, have been linked with an increased occurrence of nausea and vomiting. Anesthetic agents such as sevoflurane and propofol have also been known to induce nausea and vomiting, which typically subsides within 72 hours. This might be due to the elimination of drugs from the body within that timeframe [7,26].

Cough was observed in 4% of the patients after 24 hours and in none of the patients after seven days. The incidence of epistaxis was 3% at the end of 24 hours, and none of the participants reported it during the follow-up meeting one week later. The prevalence of cough was found to be 12% in both Farsi et al. [22] and Rajab et al. [20]. The potential causes for cough and epistaxis could be trauma due to nasotracheal intubation and the use of throat packs during surgery. These packs help prevent secretions and the aspiration of dental materials. The mucosal irritation was transient, as none of the children exhibited symptoms of cough or epistaxis at the seven-day follow-up meeting [7].

Excitement was noted in 2% of the patients after 24 hours and in none of the patients after one week. The emergence of excitement post anesthesia has been documented in pediatric patients administered sevoflurane. Sevoflurane possesses a low coefficient of blood-gas solubility, facilitating rapid washout during emergence from anesthesia. Cole et al. reported an excitement incidence of 30% in the pediatric population [27]. The peak occurrence of excitement is between the ages of two and four years, mirroring the study population of the current research [7,27]. Children who initially slept in the recovery room were more likely to experience excitement, as observed in a previous study [7].

Constipation was observed in 2% of the patients within 24 hours and in none of the patients at the one-week follow-up. Diarrhea was reported in 2% of the patients after 24 hours and in none at the one-week follow-up. Gastrointestinal complications have commonly been linked to surgical medications, especially opioids, and the patient’s dietary habits [7,22,26].

Sore throat was the least frequent postoperative complication in the children in this study, presenting in 1% of the cases. This rate dropped to 0.5% at the one-week follow-up. This rate is considerably lower than the percentages reported by Zhang et al. (7.9%) [7], Holt et al. (27%) [28], and Farsi et al. (34.4%) [22]. Multiple intubation attempts and the use of double throat packs might harm the mucosa [9,26]. This might explain why 0.5% of patients reported a sore throat at follow-up. It is essential to be gentle during intubation to minimize the risk of postoperative sore throat.

The association of OHRQoL with ECC has been explored, but its effect on postoperative complications has rarely been evaluated. This is one of the first studies of its kind [5,7]. Although various scales have been used over the years to evaluate OHRQoL, we utilized the ECOHIS. ECOHIS is a validated instrument for OHRQoL where the questionnaire is specifically designed for adult caregivers to assess the effects of oral health issues and treatment experiences on the quality of life of preschool children and their families [12]. The OHRQoL was found to improve significantly in this study [13]. The CIS scores decreased from 15.7 ± 4.1 at baseline to 12.3 ± 3.9 after 24 hours and further to 7.7 ± 1.9 at the one-week recall appointment. There was a statistically significant improvement in all domains of CIS (P < 0.001). FIS scores also decreased significantly, from 9.6 ± 2.7 at baseline to 6.1 ± 2.8 after 24 hours and to 3.5 ± 2.6 at the one-week follow-up appointment. The total ECOHIS score was found to decrease significantly within a week. This evidence suggests that comprehensive treatment can bring about significant improvements in OHRQoL over a short span of time. Our results are similar to previous studies that reported improvements in OHRQoL after full-mouth rehabilitation under general anesthesia [3-6]. However, none of these studies evaluated postoperative complications [3-6], and those that did evaluate postoperative complications [7-11,20-22] did not assess OHRQoL. This uniqueness sets our results apart.

Our study had some limitations. Responses taken from parents may not be viewed as direct responses from the child patient, a common issue in pediatric surveys. The sample in this study was limited to 200 children from a single teaching hospital within a university setting. As a result, these findings might differ when compared to studies from other locations. A more extensive sample with an even distribution of procedures, anesthesia protocols, and controlled patient and parent characteristics would be ideal. Nonetheless, our study also has notable strengths. All children returned for the follow-up visit, enabling us to evaluate all postoperative complications. To the best of our knowledge, this is the first study of its kind involving Indian children.

## Conclusions

Mild postoperative complications can emerge from full-mouth rehabilitation under general anesthesia. These complications are typically limited to one day and significantly diminish within a week. Postoperative pain was identified as the most common complication both after one day and at the one-week mark. Out of the 15 complications assessed, 11 were resolved after one week. The OHRQoL showed significant improvement posttreatment. Effective preoperative communication with caregivers is vital for managing expectations and addressing concerns related to postoperative discomfort.
